# Pneumopericardium, Pneumomediastinum, and Bilateral Pneumothoraxes Following Intubation and Repositioning in a 64-Year-Old Male Patient Undergoing Thoracic Decompression and Fusion

**DOI:** 10.7759/cureus.90086

**Published:** 2025-08-14

**Authors:** Matthew J McIntyre, Kelvin Mathew, Gisele J Wakim

**Affiliations:** 1 Department of Anesthesiology, Pain and Perioperative Medicine, Jackson Memorial Hospital, Miami, USA; 2 Department of Anesthesiology, Pain and Perioperative Medicine, University of Miami Miller School of Medicine, Miami, USA

**Keywords:** barotrauma, mechanical ventilation, pneumomediastinum, pneumopericardium, pneumothorax, surgical repositioning

## Abstract

Pneumomediastinum, pneumopericardium, and pneumothorax are recognized but rare complications associated with endotracheal intubation and mechanical ventilation in the perioperative setting. The simultaneous occurrence of all three pathologies following intubation and intraoperative repositioning has not previously been described in the literature.

This case report details the presentation and management of a 64-year-old male patient with metastatic prostate cancer undergoing thoracic decompression and fusion, who developed pneumomediastinum, pneumopericardium, and bilateral pneumothoraces following endotracheal intubation and repositioning. The patient’s medical history included a prior smoking history and known bullous emphysematous changes. Induction of general anesthesia was uneventful. Following repositioning from supine to prone, the patient experienced an acute ventilatory compromise characterized by elevated peak airway pressures, reduced tidal volumes, and hypotension. The endotracheal tube (ETT) was suctioned, albuterol was delivered, and the patient returned to baseline. Shortly after, another ventilatory compromise occurred. Interventions included suctioning of the ETT, administration of albuterol, conversion to manual ventilation, and a fiberoptic examination of the ETT. Chest radiography and computed tomography angiogram identified pneumomediastinum, pneumopericardium, and bilateral pneumothoraces. The patient was transferred to the surgical intensive care unit for conservative management, including serial chest radiographs, and ultimately recovered without further cardiopulmonary complications.

The simultaneous occurrence of pneumomediastinum, pneumopericardium, and pneumothorax following prone repositioning during general anesthesia presents a previously undocumented complication pathway requiring rapid diagnosis and management. Future work should explore standardized protocols for early recognition and intervention in “can intubate, cannot ventilate” scenarios, as well as preventive ventilatory strategies in patients with underlying pulmonary pathology.

## Introduction

Pneumomediastinum, pneumopericardium, and pneumothorax are defined as air in the mediastinal structures, the pericardial cavity, and the pleural space, respectively. Each of these pathologies is recognized as a risk associated with endotracheal intubation and mechanical ventilation. However, the incidence of pneumomediastinum, pneumopericardium, and pneumothorax following intubation and mechanical ventilation in the perioperative setting is not well described in the literature [1,2]. Pneumopericardium occurring during general anesthesia is rarer than either pneumothorax or pneumomediastinum [3]. A single case report exists describing the occurrence of both pneumopericardium and pneumomediastinum following endotracheal intubation of a 27-year-old male patient who was admitted for drug overdose [1]. To the best of our knowledge, the simultaneous presentation of pneumopericardium, pneumomediastinum, and pneumothorax following endotracheal intubation has not been previously described in the literature.

## Case presentation

**Figure 1:**
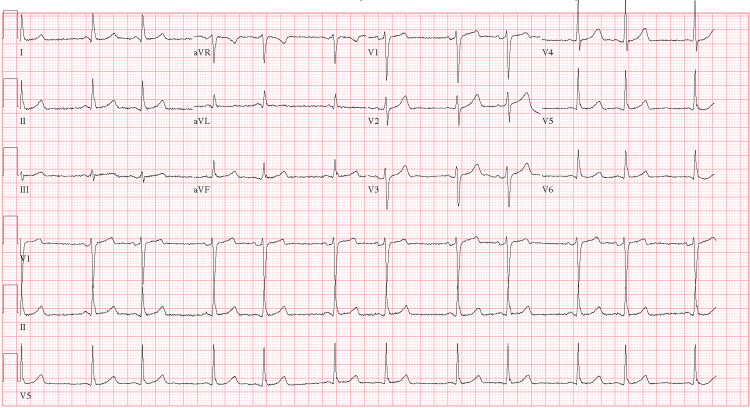
Preoperative electrocardiogram showing sinus rhythm with occasional premature ventricular complexes and premature atrial complexes aVR: augmented vector right; aVL: augmented vector left; aVF: augmented vector foot

**Figure 2:**
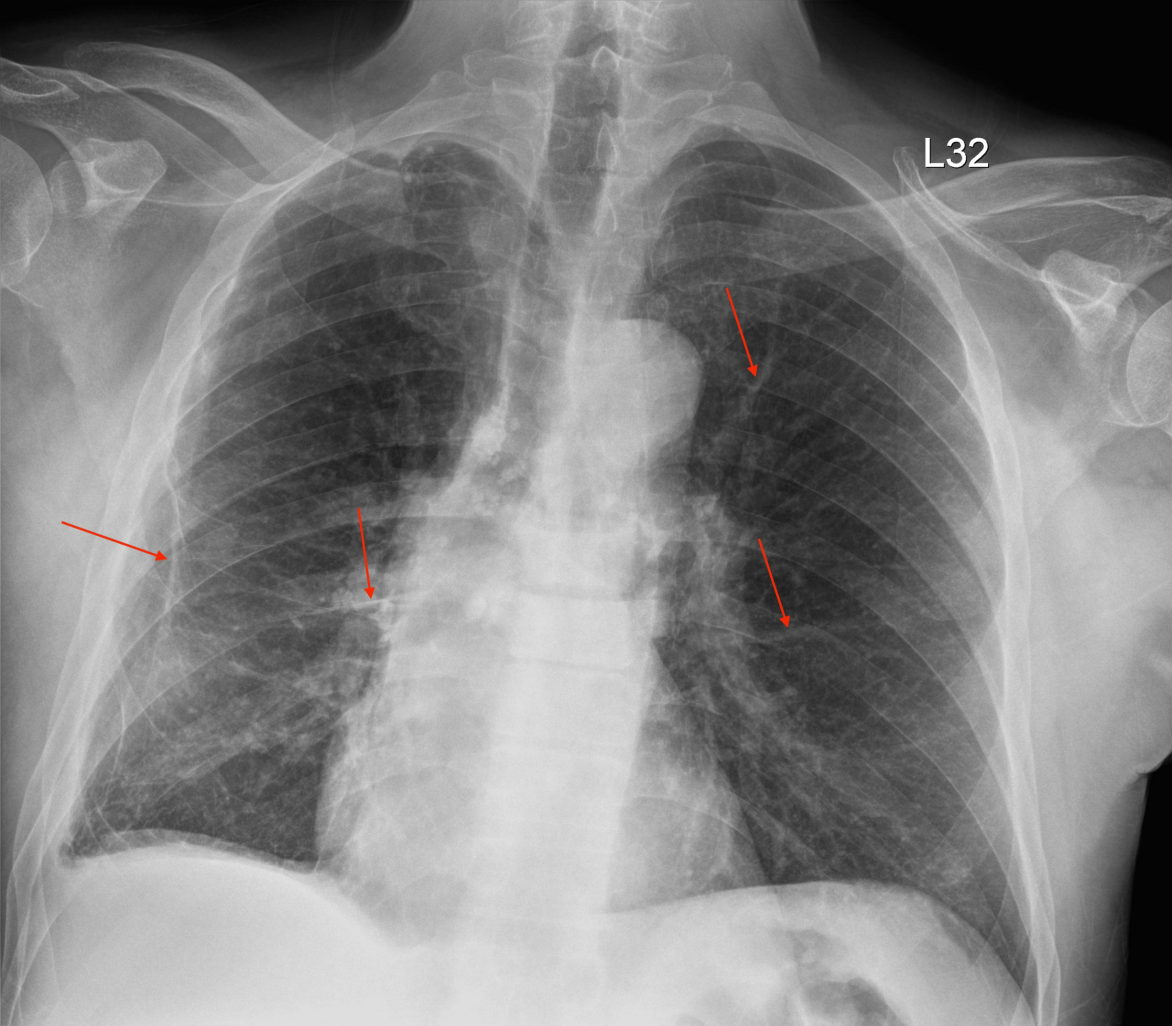
Preoperative chest X-ray Red arrows show known bullous emphysematous changes and scarring

We present the case of a 64-year-old male patient classified as American Society of Anesthesiologists IV. He is 175 cm tall and weighs 80 kg. The patient had a history of metastatic prostate cancer, which led to thoracic epidural spinal cord compression due to multiple metastases in the thoracic region. He presented for thoracic decompression and fusion. Medical history was significant for hypertension, chronic hepatitis B virus, chronic kidney disease stage 3A, prediabetes, and metastatic prostate cancer diagnosed in 2017 with prostatectomy in 2018, chemotherapy, and palliative radiation to the pelvis five months before the current presentation. He underwent cystolithopaxy in 2017 for nephrolithiasis, radical prostatectomy in 2018 for prostate cancer, and colonoscopy in 2022, with no documented previous complications with anesthesia. He had a 10-year history of smoking, but recently quit several months before the current presentation. Medications included amlodipine 10 mg daily for hypertension, duloxetine 30 mg twice daily for depression, entecavir 0.5 mg for hepatitis B, enoxaparin 40 mg daily for deep vein thrombosis prophylaxis, rosuvastatin 20 mg daily, and OxyContin 30 mg every 12 hours. The patient reported good exercise tolerance, exceeding four metabolic equivalents prior to the development of spinal metastasis. Preoperative lab results are shown in Table 1. Preoperative electrocardiogram (EKG) showed sinus rhythm with occasional premature ventricular complexes and premature atrial complexes (Figure 1). Chest X-ray showed known bullous emphysematous changes and scarring, with an unremarkable cardiomediastinal silhouette, no pulmonary edema, no pneumothorax, and no pleural effusion (Figure 2).

**Table 1:** Preoperative lab values WBC: white blood cell count; HgB: hemoglobin; Hct: hematocrit; Plt: platelets; Na+: sodium; K+: potassium; CO_2_: bicarbonate; Cl^-^: chloride; BUN: blood urea nitrogen; Cr: creatinine; Glu: glucose; PT: prothrombin time; aPTT: activated partial thromboplastin time; INR: international normalized ratio

Parameter	Preoperative value	Reference range	Unit
WBC	7.6	4-11	×10³/µL
HgB	10.6	12-16	g/dL
Hct	33.6	36-44	%
Plt	260	150-400	×10³/µL
Na⁺	136	135-145	mEq/L
K⁺	5.8	3.5-5	mEq/L
CO_2_	22	22-29	mEq/L
Cl^-^	104	95-105	mEq/L
BUN	41	7-20	mg/dL
Cr	1.4	0.6-1.3	mg/dL
Glu	112	<140	mg/dL
PT	14.1	11-13.5	seconds
aPTT	30	25-35	seconds
INR	1.06	0.8-1.2	Ratio

We proceeded with general anesthesia with a size 7.5 endotracheal tube (ETT) using the inhaled anesthetic sevoflurane and standard induction using midazolam, fentanyl, lidocaine, propofol, and rocuronium. An arterial line was placed due to the patient's increased risk of hemodynamic instability during major spinal surgery. Standard monitors were used, including noninvasive blood pressure, pulse oximetry, temperature probe, end-tidal CO_2_, and EKG. Bispectral index (BIS Quatro, Medtronic, Minneapolis, MN) was also used to monitor anesthetic depth. The patient was induced and intubated without issue. After the arterial line was placed, urology was consulted for a difficult catheter placement. Following urology’s placement of the Foley catheter using cystoscopy with a guidewire, the patient was moved to the prone position for surgical preparation of his back.

Several minutes after the patient was flipped prone, the patient became difficult to ventilate, with peak pressures reaching 37 cm H_2_O, mean airway pressure reaching 27 cm H_2_O, and blood pressure dropping from 126/84 to 67/40 mmHg with a mean arterial pressure of 51 mmHg. Inspired tidal volumes dropped from 500 to 70 mL. The ETT was suctioned, and 900 mcg of albuterol was delivered through the ETT. The patient quickly recovered, with normal tidal volumes and airway pressures. Blood pressure increased to 176/87 mmHg. A few minutes later, while the patient was still in the prone position, a similar presentation occurred, with blood pressure dropping from 176/87 to 127/61 mmHg. Peak inspiratory pressures reached 37 cm H_2_O; inspiratory tidal volume fell to 143 mL. While suctioning the ETT, it was noted to be full of secretions. The patient was quickly moved to the supine position and switched to manual ventilation. Equal bilateral breath sounds with bilateral rhonchi were heard on auscultation; 900 mcg of albuterol was delivered again through the ETT. A fiberoptic examination was conducted, revealing copious secretions within the ETT. It was confirmed that the ETT was above the carina.

**Figure 3:**
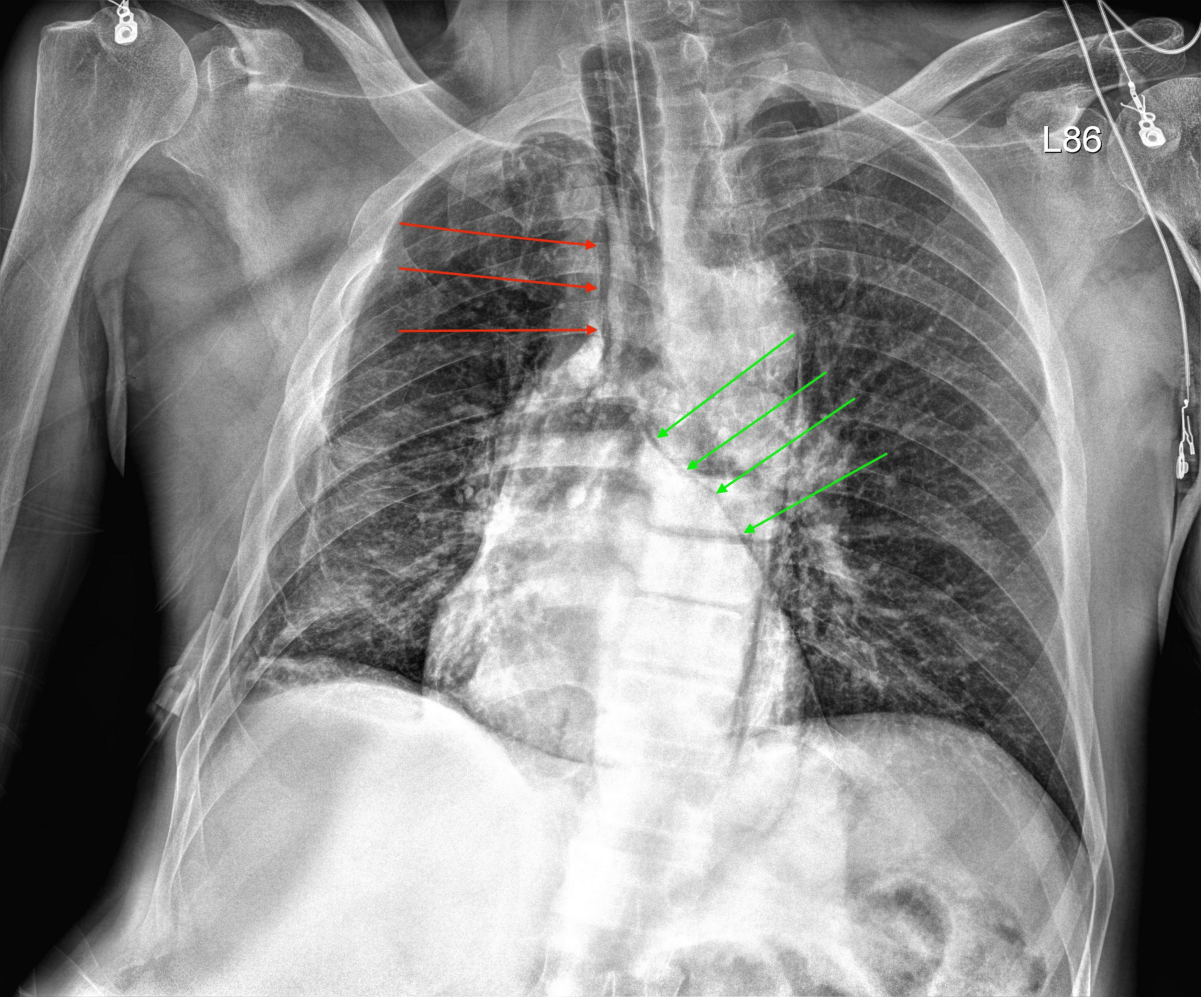
Chest X-ray taken in the operating room Red arrows show pneumomediastinum, and green arrows show pneumopericardium

**Figure 4:**
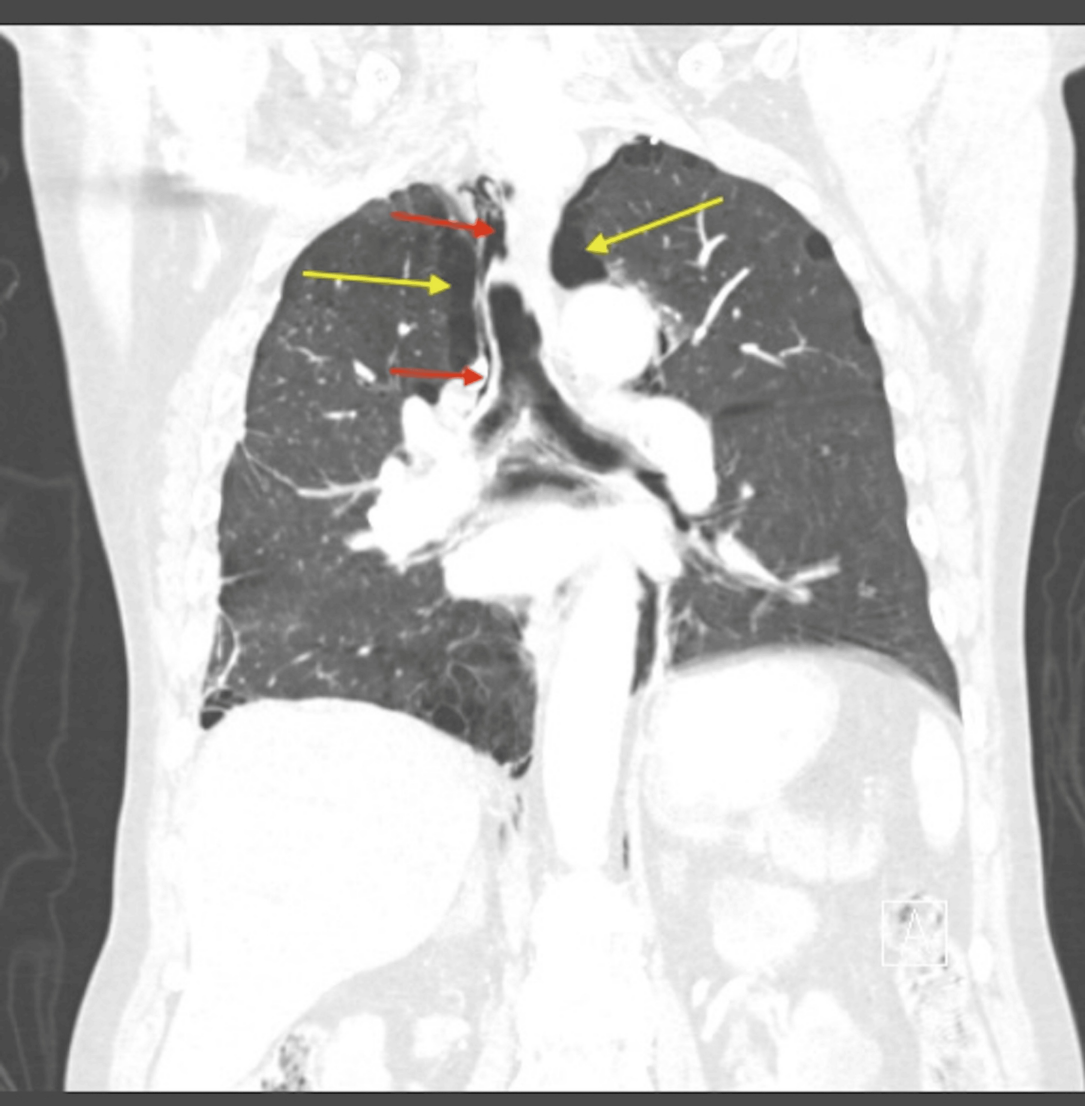
Coronal view of CT angiography showing pneumomediastinum and bilateral pneumothoraces Red arrows indicate pneumomediastinum, and yellow arrows indicate pneumothoraces CT: computed tomography

**Figure 5:**
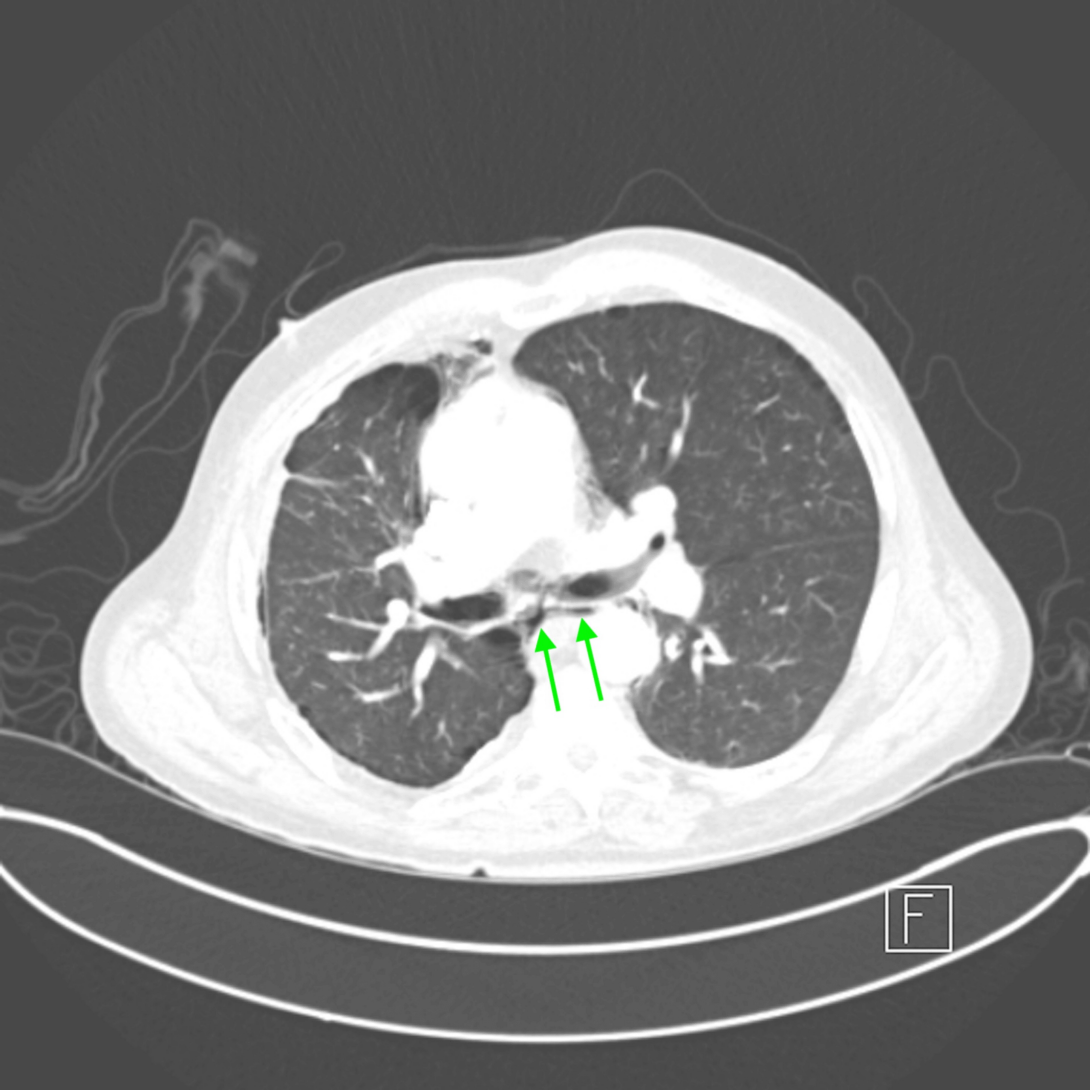
Axial view of CT angiography showing pneumopericardium Green arrows indicate pneumopericardium CT: computed tomography

**Figure 6:**
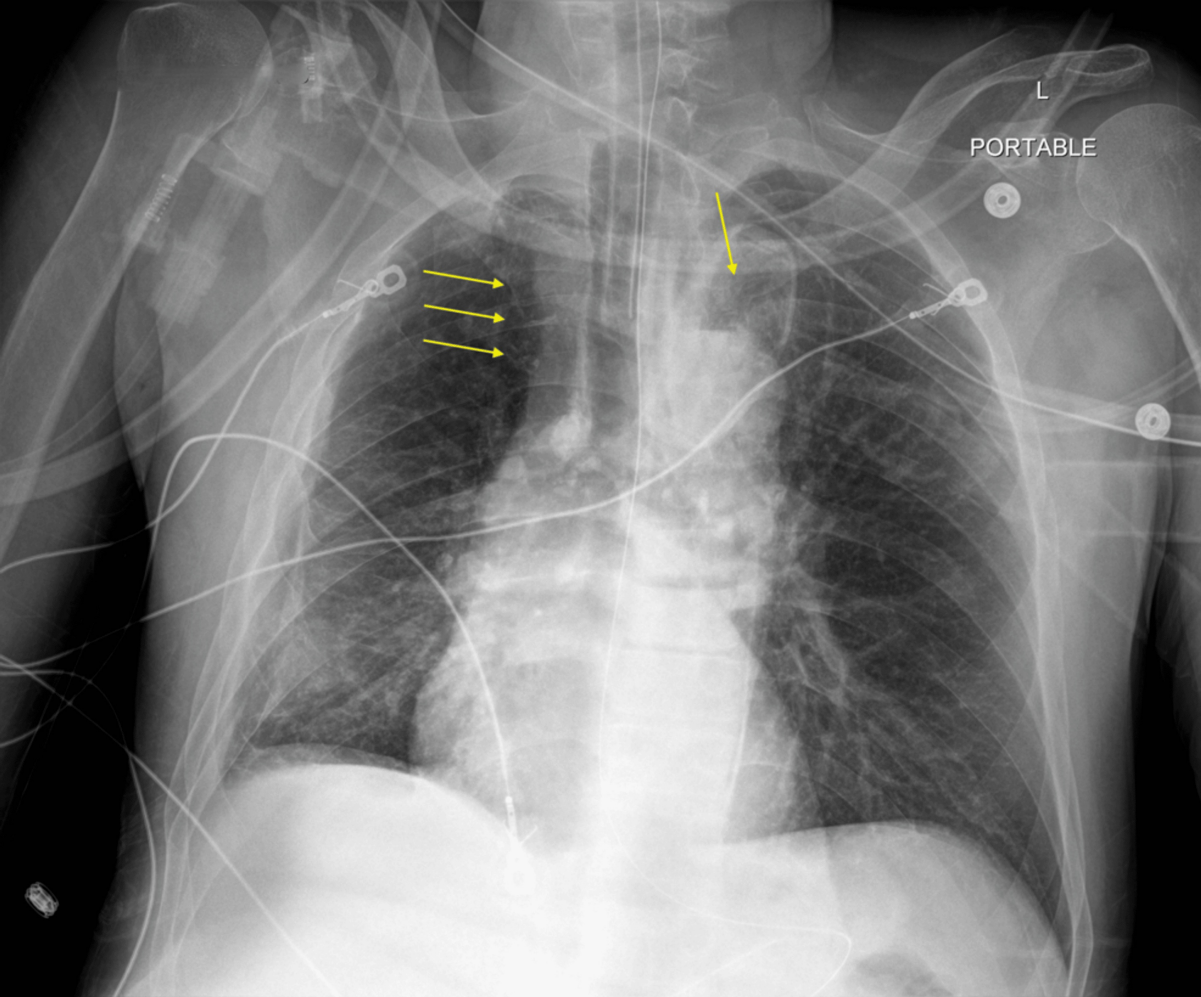
Chest X-ray 12 hours after the event Yellow arrows show expansion of the pneumothoraces

An arterial blood gas (ABG), shown in Table 2, was obtained, yielding a pH of 7.22, partial pressure of carbon dioxide of 53 mmHg, partial pressure of oxygen of 373 mmHg, and arterial concentration of bicarbonate of 22 mEq/L, with a base excess of -6, indicative of acute respiratory acidosis with hyperoxia. The ABG, along with the clinical picture, suggested an obstructive pattern of ventilation. The surgeon decided to cancel the case due to the patient being medically unstable, and the anesthesia team ordered a chest X-ray. The intraoperative chest X-ray revealed pneumomediastinum and pneumopericardium (Figure 3). The patient then underwent a computed tomography angiogram while intubated, which showed pneumomediastinum, pneumopericardium, and bilateral pneumothoraces (Figures 4, 5). He was then transported to the intensive care unit for monitoring. The cardiothoracic surgery team was consulted and recommended chest X-rays every six hours. EKG 10 hours following the event was unchanged from the preoperative EKG with normal troponins. A chest X-ray 12 hours following the event showed expansion of the pneumothoraces (Figure 6). He was extubated the following morning to room air and recovered without any further cardiopulmonary complications.

**Table 2:** Arterial blood gas PaCO_2_: arterial partial pressure of carbon dioxide; PaO_2_: arterial partial pressure of oxygen; HCO_3_^-^: arterial concentration of bicarbonate

Parameter	Intraoperative value	Reference range	Units
pH	7.22	7.35-7.45	-
PaCO_2_	53	35-45	mmHg
PaO_2_	373	80-100	mmHg
HCO₃⁻	22	22-26	mEq/L
Base excess	-6	-2 to +2	mEq/L
O₂ saturation	99.6	95-100	%

## Discussion

Pneumomediastinum, pneumopericardium, and pneumothorax are rare but known complications of perioperative anesthetic management; however, there is no documentation of the three presenting simultaneously in a patient following endotracheal intubation and repositioning. Several mechanisms describe the development of pneumomediastinum, pneumopericardium, and pneumothorax, which can be categorized as traumatic, iatrogenic, spontaneous, or secondary to underlying lung disease.

Pneumopericardium can be caused by a pleuropericardial connection secondary to chest trauma or surgical intervention [4]. Additionally, a tracheobronchial-pericardial connection can occur due to injury during intubation [1,5]. Barotrauma from mechanical ventilation can lead to pneumopericardium through the Macklin effect, in which positive pressure ventilation causes air to escape from ruptured alveoli and travel through the pulmonary perivascular sheaths into the pericardium [6]. Iatrogenic causes of pneumopericardium include pacemaker implantation, thoracic surgery, or pericardiocentesis. Less common causes of pneumopericardium include fistulas from infection, malignancy, or caustic ingestion [6].

Pneumomediastinum, like pneumopericardium, can be caused by the Macklin effect due to air escaping into the pulmonary interstitium from alveolar rupture [2]. This rupture can be caused by an increase in intrathoracic pressure resulting from severe coughing, Valsalva maneuver, or barotrauma. Barotrauma can also cause disruption of the tracheobronchial tree, allowing air to escape directly into the mediastinum [7]. Esophageal perforation from Boerhaave syndrome or intubation can lead to pneumomediastinum.

Pneumothorax is known to occur spontaneously, often in tall, thin young men without underlying lung pathology, or in adults with chronic obstructive pulmonary disease (COPD) [8]. Procedures such as central line placement, thoracentesis, or other thoracic interventions can cause pneumothorax [9]. Pneumothorax is also a known complication of mechanical ventilation. Positive pressure ventilation can cause alveolar rupture and air leakage into the pleural space [10]. In both pneumothorax and pneumopericardium, air can become trapped in the respective cavities, leading to life-threatening hemodynamic instability.

Patient positioning during surgery is also a well-known cause of pneumomediastinum, pneumopericardium, and pneumothorax [11]. Prone and Trendelenburg positioning increase intrathoracic pressure and shear stress on lung tissue, thereby increasing the likelihood of air leakage from the lungs into the mediastinum or pleural space, especially in the presence of preexisting lung pathology [11]. Additionally, obstruction of the ETT during repositioning can lead to alveolar rupture [1]. Finally, direct trauma to the airway tissue during repositioning can lead to tears in the bronchi or trachea [12].

In the case of our patient, the likely mechanism of injury was barotrauma due to an acute increase in intrathoracic pressure following repositioning, as evidenced by the increased peak inspiratory pressures of 37 cm H_2_O. ETT movement during repositioning can lead to increased airway resistance [13]. A study by Minonishi et al. showed that 91.7% of patients experienced ETT displacement during supine-to-prone transition [13]. Previous studies have shown that peak inspiratory pressures should be kept below 30 cm H_2_O in patients with COPD or emphysematous changes to the lungs to minimize the risk of barotrauma [14]. In addition to increased shear stress, prone positioning causes an increase in the elastance of the lungs, resulting in a greater required peak inspiratory pressure to maintain the same tidal volume [15]. Studies have recommended using lower tidal volumes (4-8 mL/kg) in patients with COPD/emphysematous changes to prevent volume trauma [16]. An I:E ratio of 1:3 or 1:4 is used to allow for a prolonged expiratory phase and increased autopositive end-expiratory pressure (PEEP) [14]. Ventilator PEEP is maintained at a moderate level of 5-10 cm H_2_O to prevent overinflation of distended alveoli; however, in the prone position, studies have found a PEEP of 9-12 cm H_2_O to be optimal [16]. Optimizing ventilator settings requires case-by-case adjustment based on the individual’s pulmonary resistance and compliance. Bronchospasm is another possible cause of the patient’s obstructive ventilatory pattern and ABG findings. Bronchospasm could have led to increased intrathoracic pressures, leading to his injuries.

One other possible cause of our patient’s injury was the buildup of secretions in the ETT. Occasionally, ETT secretions can accumulate and create a one-way ball-valve obstruction, in which the blockage allows air to enter the lungs during inspiration but prevents the outflow of air during expiration [17]. This can lead to barotrauma, as described in a case report where the buildup of secretions in a patient created a ball-valve effect, resulting in elevated peak inspiratory pressures and ultimately, a tension pneumothorax [18]. Given that the transient hypotension and increase in peak inspiratory pressure resolved after suctioning of the tube and delivery of albuterol, it is possible that the interventions fixed a minor ball-valve or bronchospasm that had developed.

Difficult ventilation in a patient presenting with increased peak inspiratory pressures, low tidal volumes, and hypotension requires rapid diagnosis and treatment. Although there are well-established clinical algorithms for the “cannot intubate-cannot ventilate” situation, there is no well-supported algorithm for the “can intubate-cannot ventilate” situation. A recent study by Fosse et al. describes a proposed algorithm for a “can intubate-cannot ventilate” incident [19]. The first step is to disconnect the filter from the ETT, connect the bag valve, and ventilate with 100% oxygen. If ventilation resolves, then the problem was likely related to the ventilator, hoses, or filter. The next step is to place a suction catheter through the ETT. If there is improvement, the ETT was kinked or there was an obstruction. Albuterol can also be delivered to treat potential bronchospasm. If there is still no improvement, call for help and examine the ETT with a fiberoptic scope to assess for a foreign body, obstruction, occlusion of the tube outlet, or to assess if the tube has kinked. A stethoscope should always be utilized to assess for equal and bilateral breath sounds. If, after fiberoptic scoping, no cause can be identified, there is likely a medical or surgical problem with the patient, including pneumothorax, anaphylaxis, or V/Q mismatch. A chest X-ray and ABG can help narrow the differential.

The management of pneumomediastinum, pneumopericardium, and pneumothorax depends on the severity of each. In cases of tension pneumopericardium and tension pneumothorax causing significant hemodynamic instability, immediate management involves pericardiocentesis and needle thoracostomy, followed by a tube thoracostomy, respectively. For clinically stable patients with pneumomediastinum, pneumopericardium, and pneumothorax, as in the case of our patient following initial intervention, conservative management is indicated with repeat X-rays and possible oxygen supplementation [20].

## Conclusions

Pneumomediastinum, pneumopericardium, and pneumothorax are rare complications of perioperative anesthetic management. The occurrence of these three pathologies simultaneously has not been reported in the literature. In our patient, we initially observed hypotension, increased peak inspiratory pressures, and low tidal volumes, which may have been due to bronchospasm, ETT obstruction causing a ball-valve effect, or spontaneous lung injury in a patient with emphysematous changes following repositioning. It is essential to quickly rule out pathologies when presented with an intubated patient who is difficult to ventilate. Initial steps include manually ventilating the patient, suctioning the ETT, administering a bronchodilator, and inspecting the ETT using a fiberoptic scope. It is important to call for help when initial steps show no improvement and to recognize the risks of continuing an operation with a medically unstable patient. The management of pneumomediastinum, pneumopericardium, and pneumothorax involves invasive measures if the patient is clinically unstable and conservative measures, including repeat chest X-rays and supplemental oxygen, if the patient is stable.
